# Vegetation type determines spore deposition within a forest–agricultural mosaic landscape

**DOI:** 10.1093/femsec/fiaa082

**Published:** 2020-05-01

**Authors:** Miguel A Redondo, Anna Berlin, Johanna Boberg, Jonàs Oliva

**Affiliations:** 1 Department of Forest Mycology and Plant Pathology, Swedish University of Agricultural Sciences, PO Box 7026, 750 07 Uppsala, Sweden; 2 Department of Crop and Forest Sciences, University of Lleida, Alcalde Rovira Roure 191, 25198 Lleida, Spain; 3 Joint Research Unit AGROTECNIO–CTFC, Alcalde Rovira Roure 191, 25198 Lleida, Spain

**Keywords:** dispersal limitations, high-throughput sequencing, fungal communities, community assembly, spore traps

## Abstract

Predicting fungal community assembly is partly limited by our understanding of the factors driving the composition of deposited spores. We studied the relative contribution of vegetation, geographical distance, seasonality and weather to fungal spore deposition across three vegetation types. Active and passive spore traps were established in agricultural fields, deciduous forests and coniferous forests across a geographic gradient of ∼600 km. Active traps captured the spore community suspended in air, reflecting the potential deposition, whereas passive traps reflected realized deposition. Fungal species were identified by metabarcoding of the ITS2 region. The composition of spore communities captured by passive traps differed more between vegetation types than across regions separated by >100 km, indicating that vegetation type was the strongest driver of composition of deposited spores. By contrast, vegetation contributed less to potential deposition, which followed a seasonal pattern. Within the same site, the spore communities captured by active traps differed from those captured by passive traps. Realized deposition tended to be dominated by spores of species related to vegetation. Temperature was negatively correlated with the fungal species richness of both potential and realized deposition. Our results indicate that vegetation may be able to maintain similar fungal communities across distances, and likely be the driving factor of fungal spore deposition at landscape level.

## Teaser

Vegetation type reinforces non-random patterns of fungal spore deposition.

## INTRODUCTION

The current ecological theory predicts that the composition of fungal communities is partly the result of dispersal limitations, environmental filtering and competitive exclusion (Kraft *et al*. 2015; Smith *et al*. 2018; Bruns 2019). Dispersal limitations of fungi depend on their fecundity, population size, the amount of spores released, the spore size and environmental conditions (Peay *et al*. 2012; Hussein *et al*. 2013; Norros *et al*. 2014; Peay and Bruns 2014; Dressaire *et al*. 2016; Golan and Pringle 2017). As predicted from dispersal kernels, fungal spore deposition decreases with distance from the source, leading to dispersal ranges spanning from meters (Möykkynen, von Weissenberg and Pappinen 1997; Galante, Horton and Swaney 2011; Norros *et al*. 2012; Peay *et al*. 2012; Peay and Bruns 2014) to hundreds of kilometers in the case of some pathogenic and ectomycorrhizal fungi (Brown and Hovmøller 2002; Aylor 2003; Geml *et al*. 2012). Besides distance to the source, other factors such as weather or seasonality modulate the structure of deposited spore communities (Kivlin *et al*. 2014; Fort *et al*. 2016; Mullett *et al*. 2016; Nicolaisen *et al*. 2017; Castaño *et al*. 2019). The relative importance of these factors is still under debate.

Understanding the assembly of fungal communities is important since fungi in interaction with plants play a significant role in terrestrial ecosystem functions (Clemmensen *et al*. 2013; Averill, Turner and Finzi 2014; Bennett *et al*. 2017). Determining the drivers of spore deposition can help predicting, for example, the sustainability of forest areas that are fragmented by agricultural activities or urban development (Bogar and Kennedy 2013; Mitchell *et al*. 2015; Crandall and Gilbert 2017) or the recolonization of degraded lands. The interaction between vegetation and spore dynamics can be due to different mechanisms. Vegetation patches may favor specific fungal species that may then dominate the spore communities in the air (Awad 2005). This seems to be consistent with observations at large spatial scales, where vegetation seems to be a major driver of diversity of root-associated fungi (Linde *et al*. 2018; Alzarhani *et al*. 2019; Wang *et al*. 2019) and foliar endophytes (Christian *et al*. 2016; David, Seabloom and May 2016). However, the extent to which that competitive advantage is conferred by specificity and influences the composition of deposited spores will depend on the dispersal limitations and on the distribution of the same or other types of vegetation in the landscape.

The interplay between dispersal and specificity gives rise to several scenarios. Without dispersal limitations and high specificity, we could expect small differences on deposition but differences in terms of establishment across vegetation types. However, dispersal constraints are common in fungi; therefore, differences in the composition of spore communities may already exist at the deposition stage (Peay *et al*. 2012; Peay and Bruns 2014). Furthermore, the influence of spore production should also be considered. If vegetation specificity occurs together with dispersal limitations, spore production may reinforce and further favor spatial structuring of specific fungi. Under these conditions, the fungal species favored in a certain vegetation will be amplified, and spread locally, thus diluting the spores from other vegetation types. It is possible that the same positive feedback increases the chances of local spores to reach compatible vegetation patches located far away, because the probability of a species to reach a certain distance is strongly dependent on the amount of spores at the source (Peay *et al*. 2012; Golan and Pringle 2017).

The role of vegetation on spore composition may also depend on how different fungal guilds interact with their hosts and by which mechanisms. For instance, the abundance of pathogens and endophytes seems to increase during spring and summer, most likely coinciding with the growing season of their hosts (Oliveira *et al*. 2009; Fuchs *et al*. 2017; Nicolaisen *et al*. 2017; Chen *et al*. 2018; Rather, Srinivasan and Anwar 2018). Other guilds such as saprotrophs are less active during the growing season, and their abundance may increase during autumn when they can be the primary colonizers of fresh litter (Lindahl *et al*. 2007; Santalahti *et al*. 2016). Besides vegetation, abiotic factors such as weather can also affect the diversity of fungal air communities, by influencing the production of fruiting bodies and the liberation of spores, thus affecting the number of species dispersing and being deposited at a given time point (Woo *et al*. 2018; Alzarhani *et al*. 2019). While vegetation patches are fixed in the landscape, weather occurs stochastically in space and, thus, local and regional variations in rain showers and temperature can distort spatially explicit patterns (Castaño *et al*. 2019).

Spore traps can be used to study realized and potential spore deposition. The composition of realized spore deposition can be monitored by passive traps that will capture and concentrate spores deposited by gravity or washed out from the atmosphere by precipitation (Schweigkofler, O'Donnell and Garbelotto 2004; Peay and Bruns 2014; Castaño et al. 2017, 2019). By contrast, active traps, such as volumetric air samplers, absorb spores suspended in the air, and can provide an indication of the spore potential deposition at a given site (Kivlin *et al*. 2014; Nicolaisen *et al*. 2017; Abrego *et al*. 2018; Chen *et al*. 2018; Woo *et al*. 2018). The longer the time spores remain airborne, the higher the chances that they will be absorbed by active traps. Therefore, active traps will presumably capture a greater proportion of the suspended spore community than passive traps.

The objective of this study was to determine the drivers of composition of spore communities within a forest–agricultural mosaic landscape. Several studies suggest that heterogeneous landscapes represent a good scheme to study how spore dynamics can vary across different vegetation types (Zhou and Hyde 2001; Bruns, Bidartondo and Taylor 2002; Gilbert, Gorospe and Ryvarden 2008). Our main hypothesis was that vegetation would shape the composition of deposited spore communities by amplifying local vegetation-specific species. Therefore, we expected differences in the composition of realized deposition (captured by passive traps) between different vegetation types. We anticipated that the composition of spore communities would be different for active and passive spore traps. Furthermore, we assumed that fungal guilds (i.e. different trophic interactions) would be linked to different properties of the vegetation, such as host phenology. We therefore expected that the vegetation-modulating effect on spore deposition could differ across fungal guilds.

We studied the fungal spore community in the air (potential deposition) and the deposited fungal spore community (realized deposition) by placing active and passive spore traps in three different types of vegetation (i.e. agricultural wheat fields, coniferous forests and deciduous forests) in four geographical regions in Sweden. By replicating the spore traps in a similar vegetation mosaic across four areas along a latitudinal gradient of 600 km, we could disentangle the contribution of vegetation from that of climate and distance. By placing both active and passive spore traps across different vegetation types, we could disentangle the effect of the vegetation on potential and realized deposition. In addition, by following spores weekly during the growing season (April–November) for two years (2013–2014), the role of vegetation from that of local weather events, such as temperature and precipitation, could be distinguished.

## MATERIALS AND METHODS

### Area of study and spore traps

**Figure 1.**
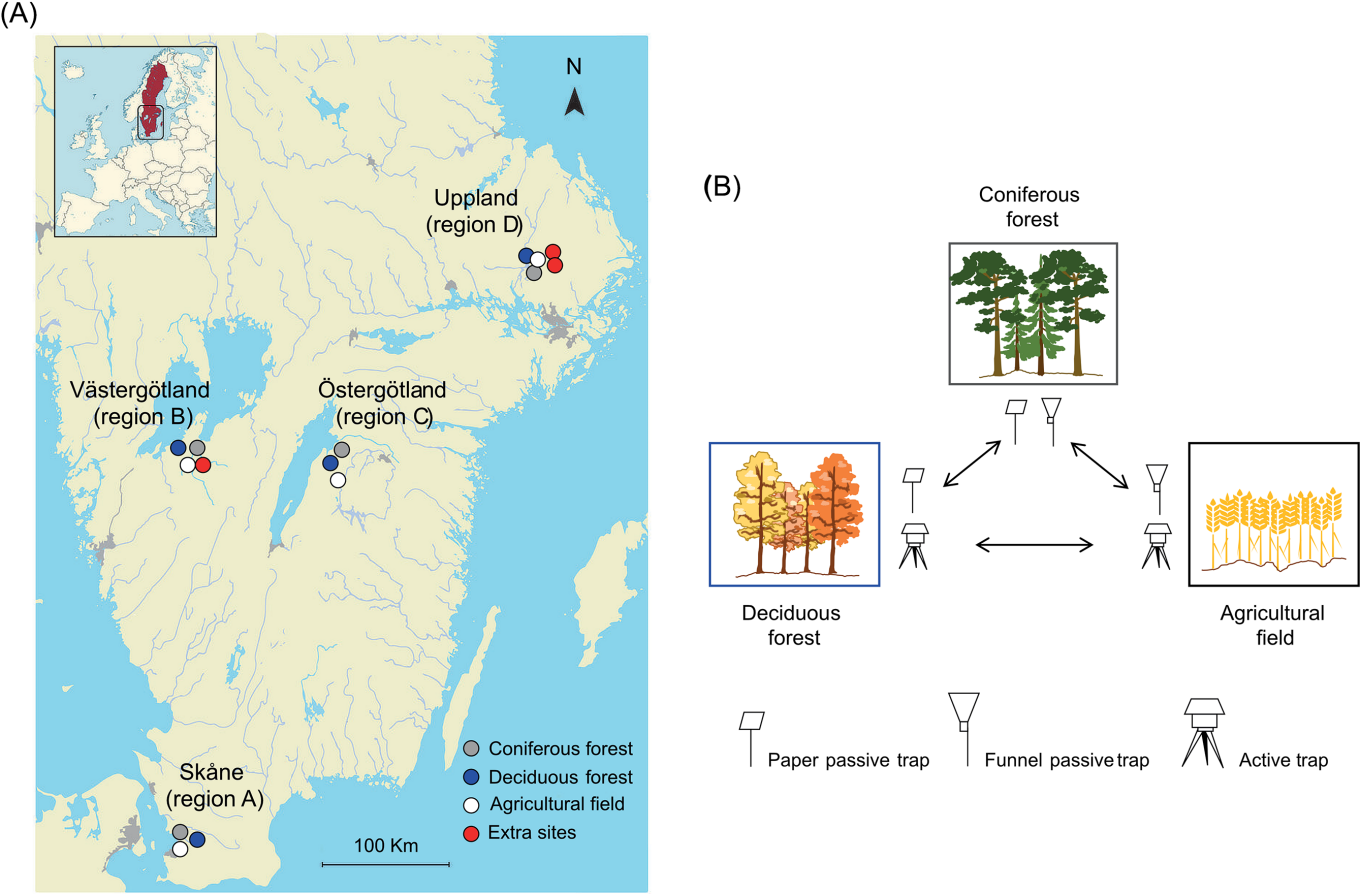
**(A)** Distribution of sampling sites in southern Sweden. **(B)** Types of spore traps placed in each type of vegetation. The arrows indicate the possible pairwise comparisons between types of vegetation for the common spore traps.

Spore traps were placed in agricultural wheat fields, coniferous forests and deciduous forests in four regions in Sweden (a total of 12 stands) (Fig. 1A; Table S1, Supporting Information). In addition to the 12 stands, one extra site of agricultural fields and each of the forest types was also sampled in regions B and D, respectively. In region B (Västergötland), we placed passive spore traps in an extra agricultural field at approximately the same distance from the sampled agricultural field and the coniferous forest nearby. The two agricultural fields were ∼6 km apart, the extra agricultural field and the coniferous forest were ∼10 km apart, and the extra agricultural field and the deciduous forest were ∼28 km apart. Similarly, in region D (Uppland), we placed passive spore traps in an extra coniferous forest and an extra deciduous forest that were approximately the same distance from the originally selected forests and fields. The extra deciduous and coniferous forests were 2.7 km apart and were located ∼15.5 km from the originally selected forests. The distances between sampling stands at each sampling region are shown in Table S2 in the Supporting Information.

We used two types of passive spore traps and one type of active trap. The two types of passive traps were a filter paper trap (hereinafter ‘paper passive trap’) and a funnel trap (hereinafter ‘funnel passive trap’). These types of traps have been used in previous studies to monitor spore dispersal (Schweigkofler, O'Donnell and Garbelotto 2004; Castaño *et al*. 2017). The paper passive traps consisted of a piece of filter paper (Whatman no. 1; 90-mm-diameter filter paper, GE Healthcare, Chicago, IL, USA) that had been soaked in 4× TE buffer as described by Schweigkofler, O'Donnell and Garbelotto (2004), dried and placed ∼1 m above the ground on a metal support. Funnel passive traps consisted of a 45-cm-diameter plastic funnel with a polysulfone filter holder containing an 8-μm nitrocellulose membrane (Merk Millipore, Burlington, MA, USA) in the end. Funnels were attached to a metal support and placed ∼1.2 m above the ground. Active traps were Ionic Spore traps (Schneider, Durr and Giles 2007) (DS Scientific, Baton Rouge, LA, USA; hereinafter ‘active trap’). In deciduous forests, active traps were placed ∼1 m above the ground. In agricultural fields, to allow a proper air flow, active traps were located on the roof of a warehouse or a tower, ∼4–8 m above ground. All active traps were programmed to be active half of the time, at 15-min intervals. The spore samples from active traps were collected on standard carbon adhesive tabs, diameter 25.4 mm (Electron Microscopy Sciences, Hatfield, PA, USA).

Traps were placed in a paired design so that the same type of spore trap would be present in two vegetation types at the time, and therefore the effect of the vegetation could be disentangled from the effect of the trap. Active traps were placed in deciduous forest and agricultural land. Passive filter paper traps were placed in coniferous and deciduous forests, and funnel traps were placed in coniferous forests and in agricultural land (Fig. 1B). Deciduous forest sites were composed of broadleaved tree species such as ash (*Fraxinus excelsior*), elm (*Ulmus* sp.), oak (*Quercus* sp.) and hazelnut (*Corylus* sp.); some forests also included beech (*Fagus sylvatica*) and alder (*Alnus* sp.). Coniferous forest sites were dominated by Scots pine (*Pinus sylvestris*) with a lesser proportion of Norway spruce (*Picea abies*). Agricultural sites consisted of wheat fields. In each type of vegetation, the two spore traps were placed 2 to 5 m from each other. The average pairwise distance between each type of vegetation in the four different regions was 12.0 km in geographical region A (Skåne), 17.7 km in region B (Västergötland), 9.5 km in region C (Östergötland) and 3.5 km in region D (Uppland). All spore trap filters were collected once per week from April to November from all sites and then sent by post to the Department of Forest Mycology and Plant Pathology at the Swedish University of Agricultural Sciences where they were stored at −20°C until DNA extraction.

### DNA extraction and library preparation

The NucleoSpin® Soil kit (Macherey-Nagel, Hoerdt, France) was used to extract DNA from half of each filter collected from the passive spore-trap types. In the case of paper passive traps, a modified version of the washing steps described by Schweigkofler, O'Donnell and Garbelotto (2004) was undertaken. Here, we used 20 ml of a different buffer containing 50 mM Tris (pH 8), 50 mM disodium ethelenediaminetetracetate (EDTA), 3% sodium dodecyl sulfate and 1 M NaCl. After the washing, 20 ml of 100% isopropanol was added and the suspension was centrifuged to concentrate the spores prior to DNA extraction. In the case of the active traps, the surface of the carbon adhesive traps was covered with 20 mg of sterile silicon dioxide to improve breaking the cell walls in the first steps of DNA extraction protocol. Amplification of the ribosomal DNA (rDNA) internal transcribed spacer 2 (ITS2) region was performed using the gITS7 (Ihrmark *et al*. 2012) and ITS4 (White *et al*. 1990) primers. The number of cycles was optimized for each sample, ranging from 20 to 33 cycles, with most samples amplifying at 27–28 cycles. Primers were tagged with unique 8-basepair tags constructed using Barcrawl software (Frank 2009). Samples were amplified in triplicates, and PCR products were purified using an AMPure kit (Beckman Counter Inc., Brea, CA, USA) and quantified with a Qubit fluorometer. Equal amounts of DNA from each sample were pooled prior to sequencing. Adaptor ligation and Pacific Biosciences SMRT PSII amplicon sequencing (hereinafter ‘PacBio’) were performed at SciLifeLab facilities [National Genomics Infrastructure (NGI), Uppsala].

### Sequence analysis and taxonomical classification

Sequences were filtered, clustered and de-multiplexed using the SCATA pipeline (http://scata.mykopat.slu.se). Sequences with an average quality score of <20, a base average quality of <3 or that lacked the primer sites (primer match value = 0.9) were removed. Sequences with different tags for the forward and the reverse primer sequences were discarded. Sequences were clustered in operational taxonomic units (OTUs) using a threshold distance of 1.5% and a penalty of one for mismatch and gap extension. OTUs with <20 reads or that appeared only once during the two-year survey were removed from the dataset. Samples with <100 reads were also discarded. Each of the OTUs were taxonomically assigned using Protax software (Somervuo *et al*. 2016; Abarenkov *et al*. 2018) in PlutoF (https://plutof.ut.ee/), using a threshold value of 0.5 (plausible classification). We chose the 0.5 probability for classification because it is considered a stricter criteria than using a 97% sequence similarity threshold (Abrego *et al*. 2018). To exclude OTUs that did not belong to the fungal kingdom, all OTUs that were not classified at phylum level by Protax were subsequently analyzed by performing a least common ancestor using the software MEGAN (Huson *et al*. 2007), with a minimum score of 300 and a minimum identity of 90%. Only the OTUs classified in MEGAN as ‘Fungi’ were retained as ‘Fungi, unknown phylum’, and merged with the OTUs previously classified by Protax.

To correct for different sample sequencing depths, we used the 15% quantile of total reads per sample as the threshold for rarefaction, which was 450 reads per sample. All samples with less reads than the 15% quantile were excluded from the analysis, and all the other samples were rarefied to 450 reads per sample using the Vegan package for R (Oksanen *et al*. 2019). Species/reads curves were obtained for each rarefied sample with the rarecurve function in the Vegan package (Figure S1, Supporting Information).

### Validation by real-time PCR

In order to validate the semi-quantitative approach of the metabarcoding analysis, the number of rarefied and non-rarefied PacBio reads of the pathogen *Hymenoscyphus fraxineus* (the causal agent of ash dieback) were compared with the number of copies obtained from quantitative real-time PCR (qPCR), as done in Castaño *et al*. (2017) with a different species. The qPCR followed the protocol described by Chandelier *et al*. (2014) and was performed using a iQ5 Multicolor Real-Time PCR Detection System (Bio-Rad Laboratories, Hercules, CA, USA). Samples were run in triplicates and each 20-µl reaction volume comprised 10 µl of PerfeCTa FastMix II for iQ (Quanta Biosciences, Beverly, MA, USA), 2 µl of MilliQ water, 1 µl of each primer and probe (final concentration 250 nM) and 5 µl of sample. PCR reactions involved an initial denaturation of 3 min at 95°C, followed by 40 cycles of 15 s at 95°C and 30 s at 60°C. Each run included a standard curve ranging from 3·10^1^ to 3·10^6^ copies/µl and two non-template controls. Standard curves were constructed by diluting a known amount of cloned PCR product quantified with a Qubit 1.0 fluorometer double stranded DNA (dsDNA) high sensitivity (HS) Assay Kit (Invitrogen, Waltham, MA, USA). Data were analyzed using iQ5 software (Bio-Rad Laboratories, Hercules, CA, USA).

### Statistical analysis

In total, 1157 samples were analyzed after rarefying (410 samples from coniferous forests, 389 samples from deciduous forests and 358 samples from agricultural fields, collected from April to November). The mean temperature and total precipitation for the week during which spores were captured were obtained from the Swedish Meteorological and Hydrological Institute website (http://www.luftweb.smhi.se). From this website, we obtained data interpolated from station data for each of the spore trap locations.

To test the relative contribution of vegetation type, week of sampling, latitude, longitude, mean temperature, and total precipitation on the composition of potential and realized deposition, we separated the dataset into two groups: the fungal communities captured by active spore traps (potential deposition) and the communities captured by passive spore traps (realized deposition). We then analyzed each group separately. Relative abundances were transformed with Hellinger transformation and then analyzed with Phyloseq (McMurdie and Holmes 2013) and Vegan package in R. A permutational multivariate analysis of variance (PERMANOVA) (adonis2 function in the Vegan package for R) was performed to obtain the variation of the spore communities that was explained by vegetation type, week of sampling, mean temperature, precipitation, latitude and longitude. To assess marginal effect of each of these terms, we added ‘by = margin’ in the arguments of the adonis2 function. Because we used two types of passive spore traps, and in order to disentangle the effect of the vegetation from the type of spore trap, we included the type of spore trap as a factor in the PERMANOVA performed on communities from passive spore traps. We then performed a principal coordinate analysis using Bray–Curtis distance to display differences in communities across vegetation types. To control for the possible effect of rare species on communities, the analysis was repeated considering only the OTUs that accounted for 80% of the total reads of the dataset. To determine differences in the relative abundance of classes of fungi across vegetation types captured by active and passive traps, OTUs were merged into the different levels of classes using the ‘tax_glom’ function in Phyloseq. OTUs belonged to a total of 41 classes. A generalized linear model was then performed on each class using the vegetation type as a fixed factor and the mean relative abundance of each class as a response. To correct for the multiple testing in these models, we used the Bonferroni correction for the 41 fungal classes identified across vegetation types (adjusted *P*-value = 0.0012). To evaluate if the realized deposition was related to vegetation specificity, we determined whether the 50 most abundant OTUs (containing ∼80% of reads) in each type of vegetation were indicator species. For this, we used the multipatt function of the indicspecies package in R (De Caceres and Jansen 2020). Because the multipatt function takes into account the relative abundance of the species, and to avoid redundance in the analysis, we transformed the count-based OTU table to presence/absence. By doing this, we could obtain which were the indicator species of each vegetation type irrespective of their abundance. To test whether the exchange of deposited spores across vegetation types was associated with a local accumulation of spores, the indicator species for each vegetation type was identified using the indicspecies package in R. For this analysis, and to avoid the potential exchange of long dispersing spores, only OTUs collected by passive spore traps were included. The correlation between the relative abundance of the indicator species in a particular vegetation type and the presence or absence of these species in the deposited spore communities of the other two vegetation types was analyzed by performing a logistic regression analysis.

To test whether fungal communities captured by active and passive spore traps were different, we analyzed samples collected from the two types of vegetation (deciduous and agricultural) in which an active and one of the two types of passive traps were placed. Coniferous forests were thus excluded from this analysis because only passive traps were placed in these stands. We performed a principal coordinate analysis for each of the two vegetation types, and we analyzed the difference between the communities obtained by each type of spore trap using the adonis2 function in R software. We obtained the Venn diagrams for each vegetation type and spore trap with the VennDiagram package in R (Chen and Boutros 2011).

To determine if weather factors were correlated with species richness, we included mean temperature and total precipitation data recorded the week before the trap filter was collected, together with the vegetation type and the year of sampling as fixed factors, and the geographical region (*n* = 4) as a random factor in a linear regression, using rarefied species richness as a response. The analysis was performed separately for each of the three types of spore traps to control for putative biases.

To test whether vegetation type and weather factors affected all types of fungi equally, we analyzed the association between types of vegetation, mean temperature, total precipitation and specific fungal guilds. OTUs were assigned to functional guild using FUNguild (Nguyen *et al*. 2016). To account for the fact that some OTUs represent more than one guild, we converted all the guilds to dummy variables so that we could assign 1 (presence) or 0 (absence) values of each guild to each OTU. To test how much of the variation of the communities of the three most common guilds (i.e. plant pathogens, wood saprotrophs and endophytes) was explained by vegetation and weather factors, we performed a PERMANOVA for each of the communities containing OTUs that were classified as plant pathogens, wood saprotrophs or endophytes. To test the association between weather factors and the relative abundance of the three most abundant guilds, the matrix of guilds and the OTU table were used as input in the FD package for R (Laliberté, Legendre and Shipley 2014) to obtain the community weighted mean (CWM) of the guild in each of the communities. Because we included the trait as binary, the value of the CWM represented the dominant guild of the community in each sample. We tested the correlation between the proportion of communities dominated by the three most common guilds and temperature. For this, we obtained the average proportion of samples dominated by each guild for each month of the survey, and then performed a regression analysis to relate these data with the average temperature of each month.

## RESULTS

### Sequencing output

After quality filtering, and before rarefying, a total of 1396 samples contained a total of 1 494 402 PacBio reads, of which 522 722 (34.9%) belonged to samples from coniferous forests, 475 289 (31.8%) belonged to samples from deciduous forests and 496 391 reads (33.2%) belonged to samples from agricultural fields. The mean number of reads per sample was 1070 [90% CI = (373–1763)]. The total number of OTUs was 2099. Samples from coniferous forests had an average number of OTUs of 144, samples from deciduous forests had an average of 107 OTUs and samples from agricultural had an average of 113 OTUs. After rarefaction, samples from coniferous forests had an average of 95 OTUs, samples from deciduous forests had an average of 70 OTUs, and samples from agricultural had an average of 74 OTUs.

### Differences in composition of deposited spore communities across vegetation types and time

**Figure 2.**
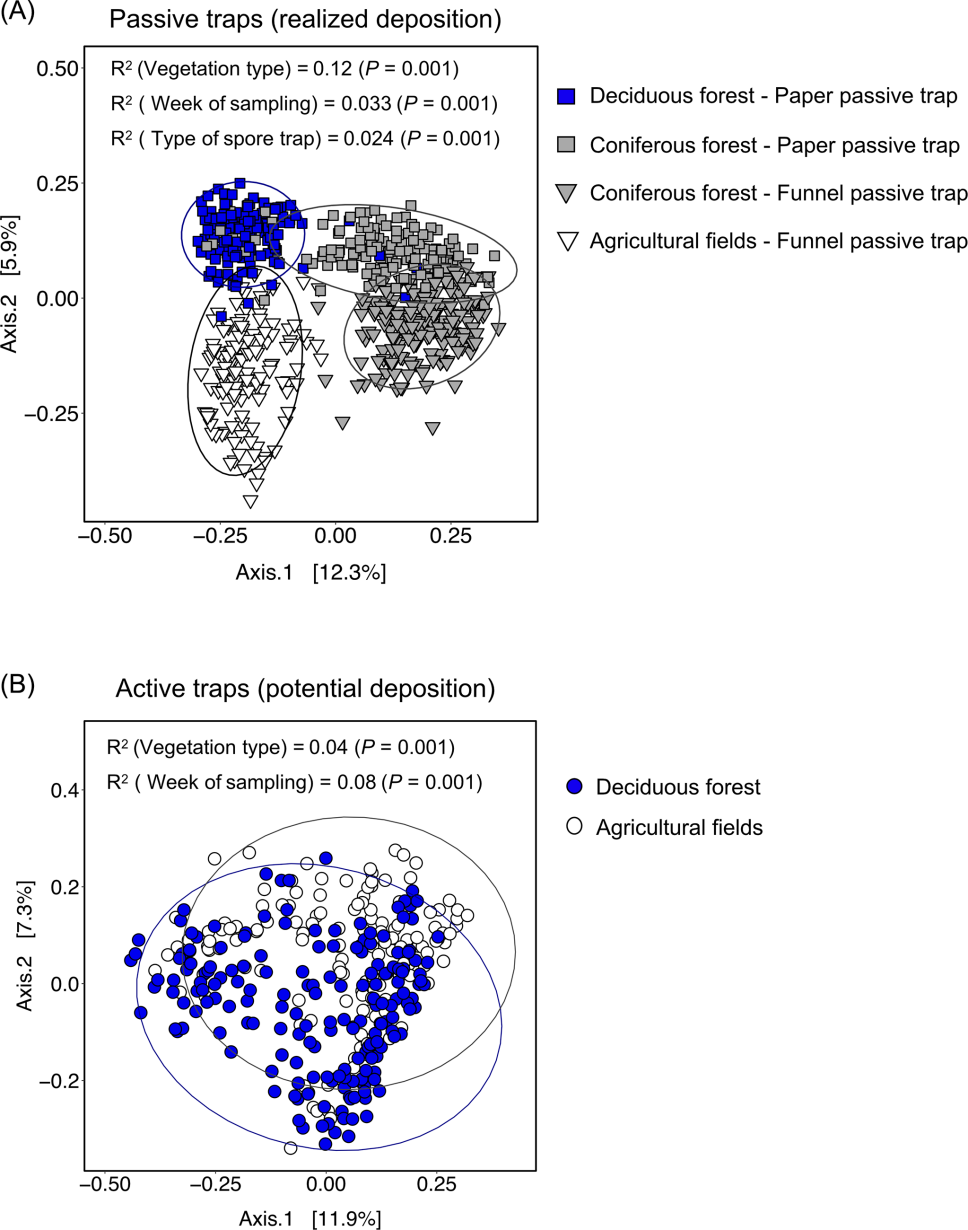
Ordination plots of a principal coordinate analysis of the spore communities collected by **(A)** passive spore traps and **(B)** active spore traps. The 95% confidence ellipse is shown for each group. The *R*^2^ and *P*-values are based on a PERMANOVA analysis performed with the adonis2 function in R.

The composition of the deposited spore communities obtained from passive traps was highly structured by the different types of vegetation (Fig. 2A). The variation explained by vegetation type was three times higher for realized deposition obtained from passive traps than for potential deposition obtained from active traps (*R*^2^ of ‘vegetation’ in realized deposition = 0.12; *R*^2^ of ‘vegetation’ in potential deposition = 0.04; Fig. 2). Furthermore, the type of vegetation contributed three times more to the variation of the communities of deposited spores than other factors such as the type of spore trap, the week of sampling or latitude (Table S3, Supporting Information). The same pattern was observed when performing pairwise comparisons between the vegetation types, for each of the passive trap types that they had in common (Figure S2, Supporting Information). By contrast, in active traps, the sampling week explained more of the variation than the type of vegetation in which the trap was placed (Fig. 2B).

When the analysis was performed on a subset of OTUs that accounted for 80% of the reads, vegetation alone explained 16.2% of the variation of the realized deposition vs 5.3% of the variation of the potential deposition (Figure S3 and Table S3, Supporting Information). The contribution of vegetation to spore deposition was consistent across spring, summer and fall for both years of the survey (Figure S4, Supporting Information). When the deposited community was analyzed separated by guilds, vegetation explained a similar amount of the variation among plant pathogens, wood saprotrophs and endophytes (*R*^2^ = 0.10, *R*^2^ = 0.095 and *R*^2^ = 0.14, respectively; Table S3, Supporting Information).

**Figure 3.**
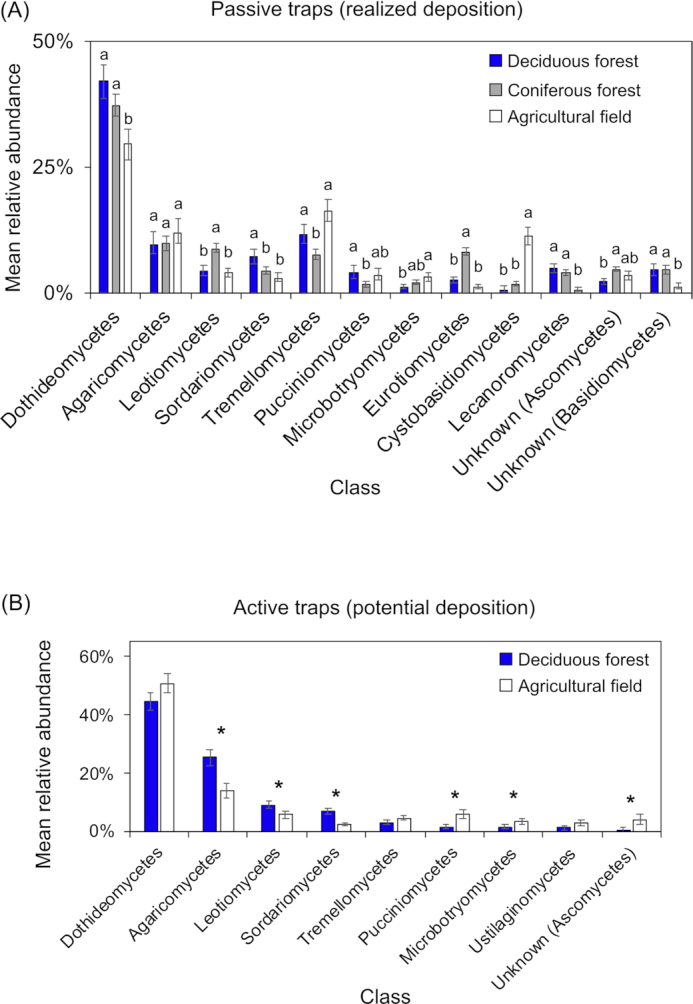
Comparison of relative abundances of fungal classes between vegetation types collected by **(A)** passive traps and **(B)** active traps. Bars indicate 95% confidence intervals. Different letters or asterisks indicate significant differences after applying the Bonferroni correction (corrected *P*-value = 0.0012).

In terms of realized deposition, Dothideomycetes, Leotiomycetes, Eurotiomycetes and Lecanoromycetes were more abundant in forests than in agricultural sites, whereas Cystobasidiomycetes were more abundant in agricultural sites (Fig. 3A). In terms of potential deposition, a higher relative abundance of Agaricomycetes, Leotiomycetes and Sordariomycetes was recorded in deciduous forests than in agricultural fields, whereas a higher relative abundance of Pucciniomycetes and Microbotryomycetes was recorded in agricultural fields than in deciduous forests (Fig. 3B).

We identified 292 indicator species in coniferous stands, 269 in deciduous stands and 296 in agricultural fields. In the three types of vegetation, more than half of the 50 most abundant OTUs (which contained ∼80% total reads) captured by passive traps were indicator species (58, 60 and 70% for coniferous, deciduous and agricultural sites, respectively). For active traps, these proportions were 40% for agricultural fields and 38% for deciduous forests.

Considering the OTUs that were captured uniquely by passive traps, we found 202 indicator species (124, 52 and 26 for coniferous, deciduous and agricultural sites, respectively). The probability of a coniferous forest indicator species being found in other vegetation patches was positively correlated with the relative abundance at the putative source (*P*-value < 0.001). The same pattern was not observed for indicator species of deciduous forests and agricultural fields found in other vegetation types. Seventeen of the coniferous forest indicator species were found in the other two vegetation types, whereas nine and three of the indicator species of deciduous and agricultural fields, respectively, were captured in another vegetation type.

### Differences in potential and realized deposition within vegetation types

**Figure 4.**
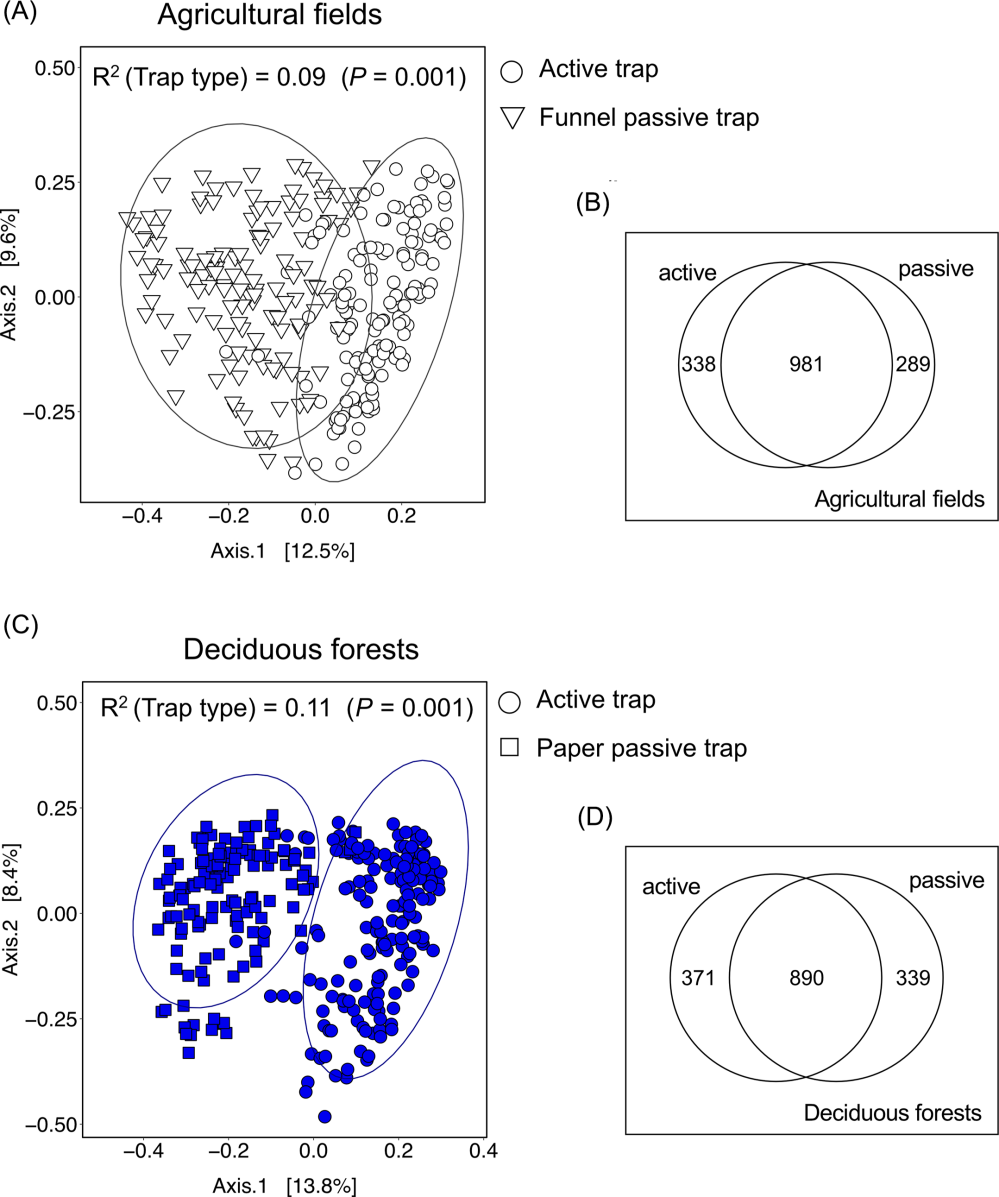
Ordination plots of a principal coordinate analysis and Venn diagrams of the spore communities collected in **(A, B)** agricultural fields and **(C, D)** deciduous forests by active and passive spore traps. The 95% confidence ellipse for each group is shown. The *R*^2^ values are based on a PERMANOVA analysis performed with the adonis2 function in R. Numbers in Venn diagrams display the number of species for each type of spore trap.

The fungal community composition of the potential deposition differed from that of the realized deposition in agricultural fields (Fig. 4A and B) and deciduous forests (Fig. 4C and D). The type of spore trap, as a proxy of potential and realized deposition, explained a similar amount of community variance in deciduous forests to that in agricultural fields (*R*^2^ deciduous forests = 0.11 vs *R*^2^ agricultural fields = 0.09). The Venn diagrams showed that 61% of the total OTUs found in agricultural fields were shared between active and passive spore traps (Fig. 4B). The overlap of OTUs between traps was of 55.6% in deciduous forests (Fig. 4D).

### Environmental factors associated with species richness

**Figure 5.**
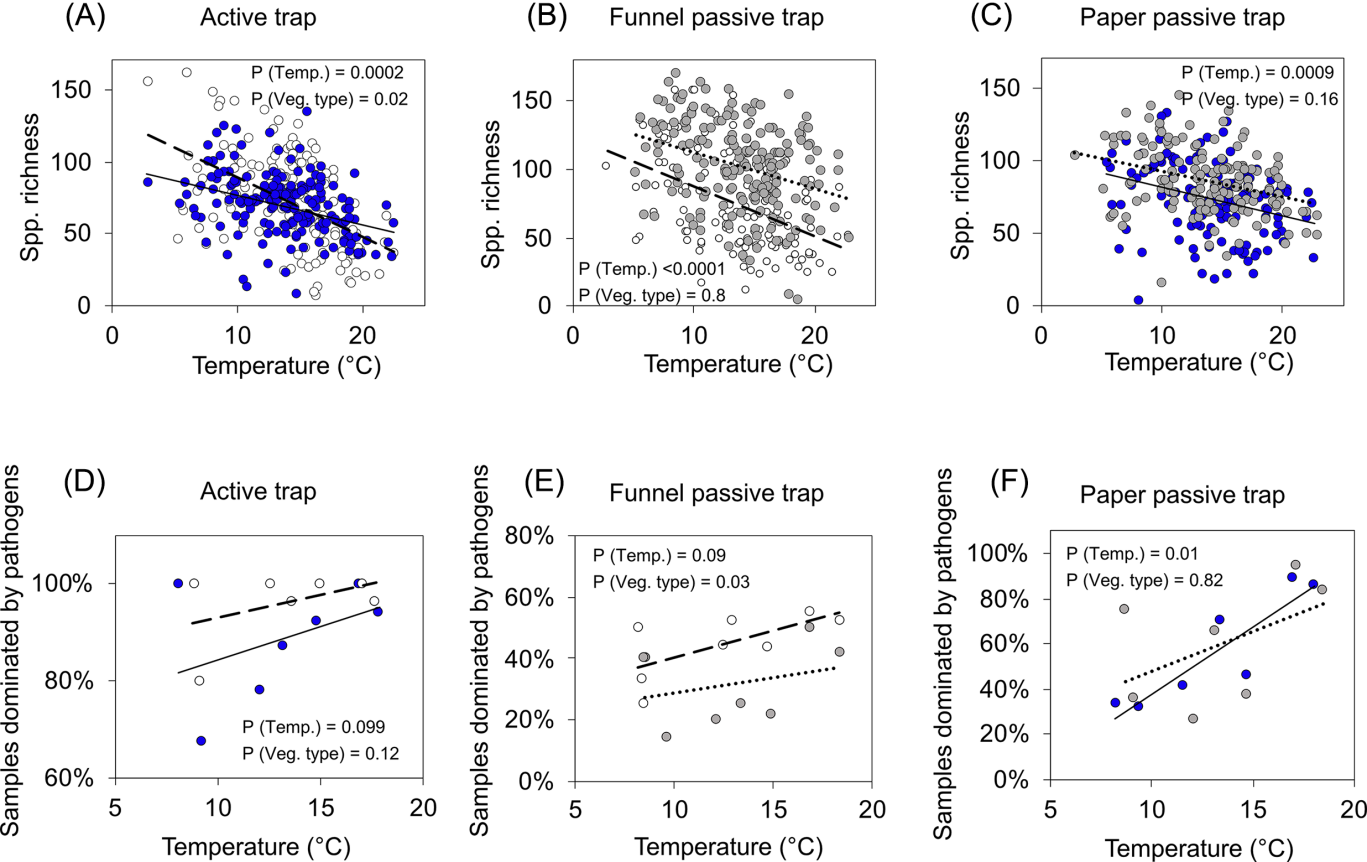
Correlation between species richness (rarefied) and temperature **(A–C)**, and between dominance of pathogens and temperature **(D–F)** for each type of spore trap and vegetation type. In panels (A–C), each dot corresponds to a sample. In panels (D–F), each dot corresponds to the averaged dominance of pathogens for each month of the survey. Blue dots and solid lines correspond to deciduous forests. White dots and dashed lines correspond to agricultural fields. Gray dots and dotted lines correspond to coniferous forests.

We found a negative correlation between temperature and species richness (Fig. 5A). Temperature explained a larger proportion of the variation in species richness than precipitation or vegetation type (Table S4, Supporting Information). The only exception was in the case of communities captured by funnel passive traps: in these communities, precipitation and temperature contributed almost equally to species richness. There was small variation between fungal guilds in terms of their response to environmental variables. However, in communities captured with paper passive traps, the dominance of plant pathogens in the fungal communities significantly increased with temperature. This pattern was marginally significant in funnel passive and active traps (Fig. 5B; Table S4, Supporting Information). The dominance of wood saprotrophs and endophytes also increased with temperature in paper passive traps (Table S4, Supporting Information).

Following the same trend found at guild level, the abundance of the plant pathogen *H. fraxineus* was positively correlated with temperature according to both PacBio and qPCR measures, with the greatest mean number of spores detected in August, followed by June and July (Figure S5a–c, Supporting Information).

## DISCUSSION

The objective of this study was to determine the factors shaping the composition of spore communities within an agriculture–forest mosaic landscape. We placed active and passive spore traps, as a proxy of the potential and realized deposition, respectively, in four sites in southern Sweden covering >600 km. As hypothesized, we found that vegetation type explained a greater proportion of the variation of deposited spore communities relative to other factors, such as weather or distance. The role of vegetation seemed to be different for potential and realized deposition. Vegetation type explained three times more of the variation of communities captured by passive traps relative to communities captured by active traps. Within each vegetation type, we found a strong differentiation between the spore communities captured by active and passive traps, suggesting that the deposited community was dominated by vegetation-specific taxa dispersing locally. Overall, our results indicate that vegetation may likely be the driving factor of fungal spore deposition at a landscape level.

Vegetation was a stronger driver of composition of deposited spore communities than distance. Across 600 km, the type of vegetation explained three times more variation of the composition of deposited spores than latitude and longitude. Across southern Sweden, composition of deposited spore communities differed more between forest and agricultural sites situated <20 km apart than across the same vegetation types separated by >100 km. By adding extra agricultural and forests sites in two sampling regions, we tested whether the differentiation due to vegetation was stronger or weaker at a local scale (within 20 km). In both regions, we found that the vegetation structured spore deposition to a similar degree as the sampled area (*R*^2^ vegetation type = 0.15 and 0.12, within the regions with extra monitoring sites, vs *R*^2^ vegetation type = 0.12 across the sampled area; Figure S6, Supporting Information). Distance decay has been suggested to affect deposition in previous studies (Bruns 2019), but according to our results, this process may be more relevant structuring deposition at a local scale than at the scales considered in our study.

Local amplification of specific taxa may be the mechanism by which vegetation shapes the composition of realized deposition. Deposited spore communities tended to be dominated by species with a lifestyle linked to the habitat where the trap was placed. For instance, the most abundant OTU deposited in coniferous forests was *Sydowia polyspora*, commonly found as an epiphyte or endophyte and also as a pathogen of conifer trees (Millberg, Boberg and Stenlid 2015). The deposited communities in agricultural fields were dominated by potential agricultural pathogens, such as *Alternaria* spp. and *Ustilago* spp. (Chen *et al*. 2018). Similarly, dominant OTUs deposited in deciduous forests were identified as *Aureobasidium pullulans* (Species hypothesis SH2149988.08FU in UNITE database) and *Diplodia* spp. (SH1143965.05FU), both previously reported in broadleaved hosts in Sweden and in other regions. Because many OTUs could not be identified at species level, we obtained the indicator species of each vegetation type, reflecting species associated with a specific vegetation. In the three types of vegetation, we found that more than half of the 50 most dominant OTUs were indicator species, again suggesting that vegetation-specific spores tended to dominate the deposited communities. This local amplification of specific spores may also be the mechanism by which vegetation structured spore deposition across large distances. In support for this, we found that higher abundances of indicator species in coniferous forests increased the chances of those species being found in other types of vegetation. We know that the distance that spores can travel depends on their abundance at the source (Golan and Pringle 2017), so it is possible that a local reinforcement of fungal species in a specific vegetation allows for colonization of distant hosts in one or several stepwise depositions (Brown and Hovmøller 2002). Alternatively, a similar deposited community might not be the result of spore exchanges between similar vegetation patches but rather be the legacy of a continuous forest landscape in the past. However, this scenario seems unlikely given the waves of deforestation and replanting that affected southern Sweden over the last centuries (Cui *et al*. 2014).

The composition of communities identified by active and passive traps was different. Although active and passive traps shared between 55 and 61% of the species, the relative proportion of the taxa was different. This was particularly evident for some of the most abundant OTUs. For instance, in deciduous stands, two species of Agaricomycetes (*Fomes fomentarius* and *Peniophora cinerea*) were among the five most dominant OTUs in active traps, but they were not within the 30 most dominant OTUs collected by passive traps. Similarly, in agricultural fields, OTUs identified as *Naganishia* spp., and *Cystobasidium* spp., were among the 10 most abundant species in passive traps, whereas none of them was within the 90 most abundant OTUs in active traps. It could be that active and passive traps capture species with different dispersal abilities. We expected that active traps would tend to capture suspended spores that are more likely to enter convective flows and be dispersed longer distances compared to spores that are deposited immediately after release. In support for this, we found that passive traps collected a greater amount of putative local taxa than active traps; passive traps collected more species with a lifestyle related to the surrounding habitat than active traps (60 and 70% indicator species within 50 most abundant OTUs in passive traps vs 40 and 38% in active traps for deciduous and agricultural sites, respectively). Since dispersal is correlated with spore size, one way to confirm that active traps captured species with long distance dispersal would have been to look at their spore size. Species with small spores such as *Peniophora* spp. (Woo *et al*. 2018) or *Ganoderma* spp. (Golan and Pringle 2017), normally regarded as long dispersers, were found more frequently in active than in passive traps. However, we were not able to test that long-distance dispersers systematically displayed small spore sizes, because most of our OTUs were not identified at species level and their spore characteristics could not be annotated. Furthermore, spore size may not be informative either since a correct annotation of dispersal ability requires the use of species-specific traps along a gradient from the source (Norros *et al*. 2012). Previous studies have mainly used one spore trap, and to the best of our knowledge, this was the first study in which different spore traps were used simultaneously. Future studies on spore dynamics may consider our trapping strategy in order to measure potential and realized deposition simultaneously.

The composition of spore communities captured by active traps was mainly shaped by seasonality. Previous studies that only used active traps also found a strong signal of seasonal variation shaping community composition (Nicolaisen *et al*. 2017; Abrego *et al*. 2018). The contribution of potential deposition on realized deposition could depend on abiotic factors such as precipitation or temperature. In our study, we found that precipitation and temperature explained more of the variation of communities captured by active traps than of those captured by passive traps. The link between spore deposition and stochastic events such as rain showers has been shown in previous studies (Woo *et al*. 2018; Castaño *et al*. 2019).

Seasonality played an important role in the abundance of plant pathogen spores. Pathogens tended to dominate the deposited spore communities during the summer months, which could be explained by a conjunction of favorable conditions in terms of spore production and host phenology. In the case of *H. fraxineus*, a specific pathogen of ash trees, qPCR data showed that its abundance was >4·10^3^ times greater in August relative to the abundance in April or October. Foliar and shoot pathogens largely rely on spores to cause infection and colonize tissue and, therefore, building up spores may be a strategy used by pathogens to establish and exclude competitors (Gladieux *et al*. 2015). A positive correlation between temperature and the abundance of pathogens, specifically rust fungi and pathogens of the genus *Ustilago*, has been reported in several studies (Oliveira *et al*. 2009; Chen *et al*. 2018). Regarding community composition, we found little variation of deposited spores of the different fungal guilds across vegetation types. It could be that the studied guilds, namely fungal pathogens, saprotrophs and endophytes, displayed a similar degree of host specificity and therefore are shaped similarly by vegetation.

Our design was set so that each spore type would be present in two vegetation types simultaneously. By doing this, we could disentangle the effect of vegetation from that of the type of spore trap. When including the type of trap as a factor in the analysis of communities obtained by passive traps across all types of vegetation, we could see that the type of vegetation explained almost three times more variation in the deposited community than the type of spore trap, weather and distance. The same pattern was observed when the analysis was performed individually for vegetation types with the same trap. Active traps in deciduous forests and agricultural fields were placed at different heights, perhaps biasing our results. However, the differences in communities between active and passive traps in deciduous forests and agricultural fields were of similar extent, indicating that the potential deposition was captured in a similar fashion in forests and fields. It is therefore unlikely that this height difference of active traps affected the overall conclusions of our study. Because of the type of traps that we used, we may have missed insect-vectored fungi. Future studies may also consider this spreading pathway when exploring patterns of fungal dispersal.

In conclusion, our study provided a comprehensive picture on how fungal communities are distributed across similar vegetation types at a landscape level. We found that the composition of deposited spore communities differs more between different types of vegetation than across distant geographical areas. We argue that the vegetation favors certain fungal taxa, allowing them to dominate spore deposition. A positive feedback of specific spores may reinforce non-random patterns of spore deposition across vegetation patches. We hypothesize that the competitive advantages of certain fungi in a specific vegetation may allow them to compensate dispersal limitations by producing large amounts of spores, thus increasing their probabilities of reaching distant hosts.

## DATA ACCESSIBILITY

The datasets of this publication, including the taxonomical classification, and the fasta file with the sequences of each OTU are archived in Figshare (DOI: https://doi.org/10.6084/m9.figshare.10012058.v5 and https://doi.org/10.6084/m9.figshare.10012058.v5).

## Supplementary Material

fiaa082_Supplemental_Files
