# Supplementary material for: Vegetation type determines spore deposition within a forest–agricultural mosaic landscape
Source: FEMS Microbiol Ecol. 2020 May 1;96(6):fiaa082. doi: 10.1093/femsec/fiaa082 (PMC7239601; doi:10.1093/femsec/fiaa082)
Supplement: fiaa082_Supplemental_Files [file fiaa082_supplemental_files.zip › Supplementary_information.docx]

**Supplementary Table S1** Location of spore traps used in this study

| Region | Site | Type of vegetation | Latitude (WGS84) | Longitude (WGS84) | Type of trap |
| --- | --- | --- | --- | --- | --- |
| Skåne (region A) |  |  |  |  |  |
|  | Dalby | Deciduous forest | 55.677003 | 13.32711 | Active trap  Paper passive trap |
|  | Lomma | Coniferous forest | 55.692293 | 13.061889 | Paper passive trap  Funnel passive trap |
|  | Alnarp | Agricultural field | 55.658775 | 13.079511 | Active trap  Funnel passive trap |
| Västergötland (region B) |  |  |  |  |  |
|  | Lanna-1 | Deciduous forest | 58.419081 | 12.771522 | Active trap  Paper passive trap |
|  | Lanna-2 | Coniferous forest | 58.4344 | 13.156259 | Paper passive trap  Funnel passive trap |
|  | Lanna-3 | Agricultural field | 58.352461 | 13.134055 | Active trap  Funnel passive trap |
|  | Extra agricultural field^a^ | Agricultural field | 58.350931 | 13.239201 | Active trap  Funnel passive trap |
| Östergötland (region C) |  |  |  |  |  |
|  | Ulvåsa | Deciduous forest | 58.551871 | 15.153068 | Active trap  Paper passive trap |
|  | Rimstad | Coniferous forest | 58.455996 | 15.182083 | Paper passive trap  Funnel passive trap |
|  | Kölbäck | Agricultural field | 58.449186 | 15.255607 | Active trap  Funnel passive trap |
| Uppland (region D) |  |  |  |  |  |
|  | Vårdsätra | Deciduous forest | 59.790296 | 17.623159 | Active trap  Paper passive trap |
|  | Lunsen | Coniferous forest | 59.776875 | 17.66609 | Paper passive trap  Funnel passive trap |
|  | Ultuna | Agricultural field | 59.816083 | 17.659735 | Active trap  Funnel passive trap |
|  | Extra deciduous forest^a^ | Deciduous forest | 59.835886 | 17.871021 | Active trap  Paper passive trap |
|  | Extra coniferous forest^a^ | Coniferous forest | 59.846903 | 17.913513 | Paper passive trap  Funnel passive trap |

^a^ Sites in which spore traps were placed in nearby additional agricultural fields and forests. These sites were not included in the overall analysis to keep the balance design. Data from the extra sites were only used in additional analysis disentangling the effect of distance and host type (Supplementary Fig. S6).

**Supplementary Table S2** Distance (Km) between the sampling sites for each region of the study.

|  |  | Region A (Skåne) | | | Region B (Västergötland) | | | | Region C (Östergötland) | | | Region D (Uppland) | | | | |
| --- | --- | --- | --- | --- | --- | --- | --- | --- | --- | --- | --- | --- | --- | --- | --- | --- |
|  |  | Dalby | Lomma | Alnarp | Lanna1 | Lanna2 | Lanna3 | Extra field | Ulvåsa | Rimstad | Kölbäck | Vårdsätra | Lunsen | Ultuna | Extra  deciduous | Extra  coniferous |
| Region A (Skåne) | Dalby | 0 | - | - |  |  |  |  |  |  |  |  |  |  |  |  |
|  | Lomma | 16.9 | 0 | - |  |  |  |  |  |  |  |  |  |  |  |  |
|  | Alnarp | 15.6 | 3.8 | 0 |  |  |  |  |  |  |  |  |  |  |  |  |
| Region B (Västergötland) | Lanna1 |  |  |  | 0 | - | - | - |  |  |  |  |  |  |  |  |
|  | Lanna2 |  |  |  | 21.25 | 0 | - | - |  |  |  |  |  |  |  |  |
|  | Lanna3 |  |  |  | 22.4 | 9.5 | 0 | - |  |  |  |  |  |  |  |  |
|  | Extra field |  |  |  | 28.2 | 10.3 | 6 | 0 |  |  |  |  |  |  |  |  |
| Region C (Östergötland) | Ulvåsa |  |  |  |  |  |  |  | 0 | - | - |  |  |  |  |  |
|  | Rimstad |  |  |  |  |  |  |  | 10.9 | 0 | - |  |  |  |  |  |
|  | Kölbäck |  |  |  |  |  |  |  | 13.1 | 4.4 | 0 |  |  |  |  |  |
| Region D (Uppland) | Vårdsätra |  |  |  |  |  |  |  |  |  |  | 0 | - | - | - | - |
|  | Lunsen |  |  |  |  |  |  |  |  |  |  | 2.8 | 0 | - | - | - |
|  | Ultuna |  |  |  |  |  |  |  |  |  |  | 3.4 | 4.4 | 0 | - | - |
|  | Extra deciduous |  |  |  |  |  |  |  |  |  |  | 15 | 14 | 12.1 | 0 | - |
|  | Extra coniferous |  |  |  |  |  |  |  |  |  |  | 16.1 | 16 | 14.6 | 2.7 | 0 |

**Supplementary Table S3** Variation of the fungal community composition of the ‘realized deposition’ (i.e., spores captured by passive traps) and ‘potential deposition’ (i.e., spores captured by active traps) explained by vegetation type, sampling time, type of spore trap, latitude, and weather factors

|  |  | All OTUs | | OTUs accounting for  80% of total reads | | OTUs corresponding to  plant pathogens | | OTUs corresponding to  wood saprotrophs | | OTUs corresponding to  endophytes | |
| --- | --- | --- | --- | --- | --- | --- | --- | --- | --- | --- | --- |
|  |  | R^2^ | *P-value* | R^2^ | *P-value* | R^2^ | *P-value* | R^2^ | *P-value* | R^2^ | *P-value* |
| Passive spore traps (realized deposition) | |  |  |  |  |  |  |  |  |  |  |
| Vegetation type | | 0.118 | **0.001**^a^ | 0.162 | **0.001** | 0.10 | **0.001** | 0.095 | **0.001** | 0.14 | **0.001** |
| Week of sampling | | 0.033 | **0.001** | 0.049 | **0.001** | 0.041 | **0.001** | 0.052 | **0.001** | 0.08 | **0.001** |
| Type of spore trap | | 0.024 | **0.001** | 0.034 | **0.001** | 0.033 | **0.001** | 0.025 | **0.001** | 0.04 | **0.001** |
| Latitude | | 0.014 | **0.001** | 0.013 | **0.001** | 0.014 | **0.001** | 0.016 | **0.001** | 0.011 | **0.001** |
| Longitude | | 0.015 | **0.001** | 0.017 | **0.001** | 0.015 | **0.001** | 0.014 | **0.001** | 0.006 | **0.002** |
| Mean temperature | | 0.017 | **0.001** | 0.023 | **0.001** | 0.027 | **0.001** | 0.044 | **0.001** | 0.036 | **0.001** |
| Total precipitation | | 0.006 | **0.001** | 0.009 | **0.001** | 0.008 | **0.001** | 0.009 | **0.001** | 0.01 | **0.001** |
| Active spore traps (potential deposition) | |  |  |  |  |  |  |  |  |  |  |
|  | Vegetation type | 0.044 | **0.001** | 0.053 | **0.001** | 0.057 | **0.001** | 0.053 | **0.001** | 0.059 | **0.001** |
|  | Week of sampling | 0.078 | **0.001** | 0.13 | **0.001** | 0.12 | **0.001** | 0.10 | **0.001** | 0.015 | **0.001** |
|  | Latitude | 0.026 | **0.001** | 0.029 | **0.001** | 0.023 | **0.001** | 0.023 | **0.001** | 0.024 | **0.001** |
|  | Longitude | 0.031 | **0.001** | 0.038 | **0.001** | 0.032 | **0.001** | 0.023 | **0.001** | 0.015 | **0.001** |
|  | Mean temperature | 0.036 | **0.001** | 0.043 | **0.001** | 0.039 | **0.001** | 0.063 | **0.001** | 0.045 | **0.001** |
| Total precipitation | | 0.01 | **0.001** | 0.013 | **0.001** | 0.013 | **0.001** | 0.013 | **0.001** | 0.01 | **0.003** |

^a^Significant *P*-values are displayed in bold.

**Supplementary Table S4** Variation of species richness and dominance of different guilds explained by type of vegetation, temperature, precipitation, and year of survey

|  |  | Species richness | | Dominance of  pathogens | | Dominance of  wood saprotrophs | | Dominance of endophytes | |
| --- | --- | --- | --- | --- | --- | --- | --- | --- | --- |
|  |  | F-value | *P* | F-value | *P* | F-value | *P* | F-value | *P* |
| Paper passive trap | |  |  |  |  |  |  |  |  |
| Mean temperature | | 11.91 | **0.0006**^a^ | 8.97 | **0.01** | 6.81 | **0.024** | 25.2 | **0.0004** |
| Vegetation type | | 2.52 | 0.11 | 0.05 | 0.82 | 0.005 | 0.94 | 1.19 | 0.3 |
| Total precipitation | | 0.78 | 0.38 | – | ­– | – | – | – | – |
| Year | | 0.32 | 0.57 | – | – | – | – | – | – |
| Vegetation type × mean temperature | | 0.0063 | 0.94 | – | – | – | – | – | – |
| Vegetation type × total precipitation | | 0.08 | 0.78 | – | – | – | – | – | – |
|  | Vegetation type × year | 0.48 | 0.49 | – | – | – | – | – | – |
|  | Mean temperature × year | 1.82 | 0.18 | – | – | – | – | – | – |
|  | Total precipitation × year | 1.51 | 0.22 | – | – | – | – | – | – |
| Funnel passive trap | |  |  |  |  |  |  |  |  |
|  | Mean temperature | 51.83 | **<0.0001** | 3.33 | 0.09 | 0.26 | 0.61 | 0.14 | 0.7 |
|  | Vegetation type | 0.0008 | 0.97 | 5.74 | **0.03** | 1.4 | 0.24 | 5.17 | **0.04** |
|  | Total precipitation | 26.4 | **<0.0001** | – | – | – | – | – | – |
|  | Year | 0.03 | 0.86 | – | – | – | – | – | – |
| Vegetation type × mean temperature | | 6.35 | **0.012** | – | – | – | – | – | – |
| Vegetation type × total precipitation | | 2.09 | 0.15 | – | – | – | – | – | – |
|  | Vegetation type × year | 1.6 | 0.21 | – | – | – | – | – | – |
|  | Mean temperature × year | 3.81 | 0.052 | – | – | – | – | – | – |
|  | Total precipitation × year | 20.5 | **<0.0001** | – | – | – | – | – | – |
| Active trap | |  |  |  |  |  |  |  |  |
|  | Mean temperature | 14.71 | **0.0002** | 3.23 | 0.099 | 0.1 | 0.75 | 4.04 | 0.07 |
|  | Vegetation type | 6.13 | **0.014** | 2.8 | 0.12 | 5.6 | 0.03 | 0.02 | 0.89 |
|  | Total precipitation | 2.19 | 0.14 | – | – | – | – | – | – |
|  | Year | 1.4 | 0.23 | – | – | – | – | – | – |
| Vegetation type × mean temperature | | 8.18 | **0.0044** | – | – | – | – | – | – |
| Vegetation type × total precipitation | | 0.03 | 0.86 | – | – | – | – | – | – |
|  | Vegetation type × year | 6.44 | **0.011** | – | – | – | – | – | – |
|  | Mean temperature × year | 0.07 | 0.79 | – | – | – | – | – | – |
|  | Total precipitation × year | 3.7 | 0.055 | – | – | – | – | – | – |

^a^ Significant *P*–values are shown in bold.

**Supplementary Figure S1** Rarefaction curve for each sample at sequencing depth of 450 reads per sample.

**Supplementary Figure S2** Ordination plots of a principal coordinate analysis of spore communities collected by (a) funnel spore traps and (b) lipless spore traps. The 95% confidence ellipse is shown for each group. The R^2^ values are based on a PERMANOVA analysis performed with the adonis2 function in R.

**Supplementary Figure S3** Ordination plots of a principal coordinate analysis of spore communities collected by (a) passive spore traps and (b) active spore traps, considering only the OTUs that accounted for 80% of the total number of reads (total OTUs = 103). The 95% confidence ellipse is shown for each group. The R^2^ values are based on a PERMANOVA analysis performed with the adonis2 function in R.

**Supplementary Figure S4** Ordination plots of a principal coordinate analysis of spore communities collected by (a) passive spore traps and (b) active spore traps for each year and season. The 95% confidence ellipse is shown for each group.

**Supplementary Figure S5** Results of qPCR of *Hymenoscyphus fraxineus*. Correlation between rarefied (a) and non–rarefied (b) reads obtained by Pacific BioSciences (PacBio) sequencing and copies of *H. fraxineus* obtained by qPCR. (c) Distribution of mean copy number of *H. fraxineus* and temperature for each month of the survey. Mean values are averaged for both years of the survey. Bars indicate the standard errors.

**Supplementary Figure S6.** Ordination plots of a principal coordinate analysis of the additional spore communities collected in (a) Västergötland (region B) and (b) Uppland (region D). (a) Red samples represent the spores captured in an extra agricultural field (agricultural field 2) located ca. 7 km away from agricultural field 1 and ca. 9 km far away from the deciduous forest. The 95% confidence ellipse is shown for each group. (b) Orange samples represent the communities from an extra coniferous forest (coniferous forest 2). Light–blue samples represent the communities from an extra deciduous forest (deciduous forest 2). The extra coniferous and deciduous forests were located 2.7 km apart from each other and ca. 15.5 km from coniferous forest 1 and deciduous forest 1. The 95% confidence ellipse is shown for each group. The R^2^ values are based on a PERMANOVA analysis performed with the adonis2 function in R.
